# Supplementary material for: Mapping qualitative research on motor imagery: A scoping review
Source: PLoS One. 2026 Apr 29;21(4):e0348064. doi: 10.1371/journal.pone.0348064 (PMC13127901; doi:10.1371/journal.pone.0348064)
Supplement: S1 File — Complete search strategy, developed in collaboration with Health Sciences librarian for all databases searched. (PDF) [file pone.0348064.s001.pdf]

CINAHL [EBSCOhost]		
**Search ID**	**Search Terms**	**Results**
S1	(MH "Qualitative Studies+") OR (MH "Case Studies")	231,238
S2	((qualitative OR exploratory OR narrative OR interpretive OR ethnograph* OR phenomenology* OR case OR grounded OR mixed OR multimethod OR multi-method) N3 (research OR stud* OR inquir* OR method* OR investigation OR design OR theory OR descripti*)) OR (when N5 what OR when N5 where OR when N5 why OR when N5 who OR where N5 what OR where N5 why OR where N5 who OR what N5 why OR what N5 who OR why N5 who)) OR qualitative)	593,311
S3	S1 OR S2	611,456
S4	((motor OR mental OR movement OR action OR kinesthetic OR kinaesthetic OR athlet* OR sport* OR dance*) N3 (imagery OR rehearsal OR simulation OR visuali*))	2,571
S5	S3 AND S4	334

SPORTDiscus [EBSCOhost]		
**Search ID**	**Search Terms**	**Results**
S1	DE "MOTOR imagery (Cognition)"	103
S2	((qualitative OR exploratory OR narrative OR interpretive OR ethnograph* OR phenomenology* OR case OR grounded OR mixed OR multimethod OR multi-method) N3 (research OR stud* OR inquir* OR method* OR investigation OR design OR theory OR descripti*)) OR (when N5 what OR when N5 where OR when N5 why OR when N5 who OR where N5 what OR where N5 why OR where N5 who OR what N5 why OR what N5 who OR why N5 who)) OR qualitative)	78,015
S3	((motor OR mental OR movement OR action OR kinesthetic OR kinaesthetic OR athlet* OR sport* OR dance*) N3 (imagery OR rehearsal OR simulation OR visuali*))	2,977
S4	S1 OR S3	2,977
S5	S2 AND S4	194

PsychInfo [EBSCOhost]		
**Search ID**	**Search Terms**	**Results**
S1	(((DE "Qualitative Methods" OR DE "Coding Scheme" OR DE "Content Analysis" OR DE "Ethnography" OR DE "Focus Group" OR DE "Grounded Theory" OR DE "Interpretative Phenomenological Analysis" OR DE "Narrative Analysis" OR DE "Semi-Structured Interview" OR DE "Thematic Analysis") OR (DE "Mixed Methods Research")) OR (DE "Narratives")) OR (DE "Phenomenology")	86,370
S2	((qualitative OR exploratory OR narrative OR interpretive OR ethnograph* OR phenomenology* OR case OR grounded OR mixed OR multimethod OR multi-method) N3 (research OR stud* OR inquir* OR method* OR investigation OR design OR theory OR descripti*)) OR (when N5 what OR when N5 where OR when N5 why OR when N5 who OR where N5 what OR where N5 why OR where N5 who OR what N5 why OR what N5 who OR why N5 who)) OR qualitative)	593,633
S3	S1 OR S2	632,826
S4	((motor OR mental OR movement OR action OR kinesthetic OR kinaesthetic OR athlet* OR sport* OR dance*) N3 (imagery OR rehearsal OR simulation OR visuali*))	10,381
S5	S3 AND S4	916

MEDLINE [Ovid]		
**Search ID**	**Search Terms**	**Results**
1	exp qualitative research/	105,390
2	exp grounded theory/	3342
3	1 or 2	106959
4	(((qualitative or exploratory or narrative or interpretive or ethnograph* or phenomenolog* or case or grounded or mixed or multimethod or multi-method) adj3 (research or stud* or inquir* or method* or investigation or design or theory or descripti*)) or ((when adj5 what) or (when adj5 where) or (when adj5 why) or (when adj5 who) or (where adj5 what) or (where adj5 why) or (where adj5 who) or (what adj5 why) or (what adj5 who) or (why adj5 who)) or qualitative).mp.	1,236,658
5	3 or 4	10,149
6	((motor or mental or movement or action or kinesthetic or kinaesthetic or athlet* or sport* or dance*) adj3 (imagery or rehearsal or simulation or visuali*)).mp.	10,149
7	5 and 6	578

Embase [Elsevier]

Search ID	Search Terms	Results
#1	**'qualitative research'**/exp OR **'ethnography'**/exp OR **'phenomenology'**/exp OR **'grounded theory'**/exp OR **'narrative'**/exp OR **'thematic analysis'**/exp OR **'content analysis'**/exp OR **'interpretive phenomenological analysis'**/exp OR **'mixed methods study'**/exp OR **'mixed methods'**/exp	241,214
#2	**'motor imagery'**/exp OR **'motor imagery training'**/exp	824
#3	((**qualitative** OR **exploratory** OR **narrative** OR **interpretive** OR **ethnograph*** OR **phenomenology*** OR **case** OR **grounded** OR **mixed** OR **multimethod** OR **'multi-method'**) NEAR/3 (**research** OR **stud*** OR **inquir*** OR **method*** OR **investigation** OR **design** OR **theory** OR **descripti***)) OR (**when** NEAR/5 **what**) OR (**when** NEAR/5 **where**) OR (**when** NEAR/5 **why**) OR (**when** NEAR/5 **who**) OR (**where** NEAR/5 **what**) OR (**where** NEAR/5 **why**) OR (**where** NEAR/5 **who**) OR (**what** NEAR/5 **why**) OR (**what** NEAR/5 **who**) OR (**why** NEAR/5 **who**) OR **qualitative**	1,522,099
#4	(**motor** OR **mental** OR **movement** OR **action** OR **kinesthetic** OR **kinaesthetic** OR **athlet*** OR **sport*** OR **dance***) NEAR/3 (**imagery** OR **rehearsal** OR **simulation** OR **visuali***)	12,332
#5	#2 OR #4	12,332
#6	#1 OR #3	1,572,420
#7	#5 AND #6	726

Scopus [Elsevier]		
**Search ID**	**Search Terms**	**Results**
1	TITLE-ABS-KEY (((qualitative OR exploratory OR narrative OR interpretive OR ethnograph* OR phenomenolog* OR "case study" OR "grounded theory" OR "mixed methods" OR multimethod OR "multi-method") W/3 (research OR study OR studies OR inquiry OR inquiries OR method* OR investigation OR design OR theory OR description)) OR (("when" W/5 "what") OR ("when" W/5 "where") OR ("when" W/5 "why") OR ("when" W/5 "who") OR ("where" W/5 "what") OR ("where" W/5 "why") OR ("where" W/5 "who") OR ("what" W/5 "why") OR ("what" W/5 "who") OR ("why" W/5 "who")) OR qualitative)	2,919,374
2	TITLE-ABS-KEY ((motor OR mental OR movement OR action OR kinesthetic OR kinaesthetic OR athlet* OR sport* OR dance*) W/3 (imagery OR rehearsal OR simulation OR visuali*))	39,510
3	1 AND 2	1,807

Dissertations and Theses Global [ProQuest] Attempt #1		
**Search ID**	**Search Terms**	**Results**
S1	abstract(((qualitative OR exploratory OR narrative OR interpretive OR ethnograph* OR phenomenolog* OR "case study" OR "grounded theory" OR "mixed methods" OR multimethod OR "multi-method") NEAR/3 (research OR study OR studies OR inquiry OR inquiries OR method* OR investigation OR design OR theory OR description)) OR (("when" NEAR/5 "what") OR ("when" NEAR/5 "where") OR ("when" NEAR/5 "why") OR ("when" NEAR/5 "who") OR ("where" NEAR/5 "what") OR ("where" NEAR/5 "why") OR ("where" NEAR/5 "who") OR ("what" NEAR/5 "why") OR ("what" NEAR/5 "who") OR ("why" NEAR/5 "who")) OR qualitative) OR title(((qualitative OR exploratory OR narrative OR interpretive OR ethnograph* OR phenomenolog* OR "case study" OR "grounded theory" OR "mixed methods" OR multimethod OR "multi-method") NEAR/3 (research OR study OR studies OR inquiry OR inquiries OR method* OR investigation OR design OR theory OR description)) OR (("when" NEAR/5 "what") OR ("when" NEAR/5 "where") OR ("when" NEAR/5 "why") OR ("when" NEAR/5 "who") OR ("where" NEAR/5 "what") OR ("where" NEAR/5 "why") OR ("where" NEAR/5 "who") OR ("what" NEAR/5 "why") OR ("what" NEAR/5 "who") OR ("why" NEAR/5 "who")) OR qualitative)	600,703
S2	abstract(((motor OR mental OR movement OR action OR kinesthetic OR kinaesthetic OR athlet* OR sport* OR dance*) NEAR/3 (imagery OR rehearsal OR simulation OR visuali*))) OR title(((motor OR mental OR movement OR action OR kinesthetic OR kinaesthetic OR athlet* OR sport* OR dance*) NEAR/3 (imagery OR rehearsal OR simulation OR visuali*)))	3,694
S3	[S1] AND [S2]	378
