# Supplementary material for: Mapping qualitative research on motor imagery: A scoping review
Source: PLoS One. 2026 Apr 29;21(4):e0348064. doi: 10.1371/journal.pone.0348064 (PMC13127901; doi:10.1371/journal.pone.0348064)
Supplement: S2 File — Definitions of all exclusion criteria applied during the full-text screening stage. (PDF) [file pone.0348064.s002.pdf]

Exclusion Criteria:

- **Not MI:** The study does not investigate motor imagery. The type of mental imagery described is not specifically motor in nature.

- **Not exclusively MI:** The study includes motor imagery but combines it with other interventions (e.g., action observation, imitation, virtual reality).

- **Main aim not qual MI:** The study includes both motor imagery and qualitative methods, but the qualitative component is not focused on motor imagery (i.e., it focuses on intervention effectiveness or participant experience more broadly). Alternatively, motor imagery and qualitative methods are present, but not the primary focus or research aim.

- **No full text available:** The full text of the study is unavailable, either because the study is incomplete or cannot be accessed.

- **Non-English language:** The study is not published in English.

- **Not qualitative:** The study does not use qualitative methods to address its research questions.

- **Not a research study:** The article does not report original research or a systematic/scoping review of original research.

- **Duplicate – thesis version of published manuscript:** The source is a thesis that duplicates a study already published in a peer-reviewed journal.
