# Supplementary material for: Mapping qualitative research on motor imagery: A scoping review
Source: PLoS One. 2026 Apr 29;21(4):e0348064. doi: 10.1371/journal.pone.0348064 (PMC13127901; doi:10.1371/journal.pone.0348064)
Supplement: S3 File — Data extraction form and accompany instructions used in the data extraction stage. (PDF) [file pone.0348064.s003.pdf]

GENERAL INFORMATION

Study ID:
Reviewers will assign a unique study ID to every source to be extracted. Begin with **S01** and continue sequentially (e.g., S02, S03, ...) for each new study. Ensure that no duplicate IDs are used.

Title:
Title of source. Write the full title using sentence case.

Example: Lived experiences of physical therapists treating traumatic knee injury using integrated psychological training (MOTIFS): A qualitative interview study.

(Notes: Capitalize first letter following colon. Acronyms in uppercase)

Author(s):

- *One author:* Smith
- *Two authors:* Smith & Hunt
- *Three or more authors:* Smith et al.

Affiliation of Primary Author:
Record the full institutional affiliation of the **first author** as stated in the published article.
Example: Department of Health Sciences, Lund University, Lund, Sweden.

For theses/dissertations, report the **faculty (if specified)** and **university** as listed on the **title page.**
Example: Faculty of the School of Professional Studies of Gonzaga University.

Publication:
Indicate where the article was published. For journal articles, write the *full journal name* in **Title Case** (capitalize major words). If the source is a thesis, enter **ProQuest Dissertations and Theses** as the publication name.

Full journal name examples:

- Physiotherapy Theory and Practice
- BMJ Open Sport and Exercise Medicine
- Journal of Sports Sciences

Year:
Record the year the article was published shown on the full-text version uploaded to Covidence.

Country of origin:

- Africa
    - e.g., South Africa, Nigeria, Kenya, Egypt, Ethiopia

- Asia: Includes South, East, Southeast, and Central Asia
    - e.g., China, India, Japan, Indonesia, Pakistan, South Korea

- Middle East
    - e.g., Iran, Israel, Saudi Arabia, Turkey, United Arab Emirates

- Europe
    - e.g., United Kingdom, Germany, France, Italy, Poland, Sweden

- North America
    - Canada
    - United States
    - Mexico

- Latin America & Caribbean: Includes Central America, South America, and the Caribbean
    - e.g., Brazil, Argentina, Colombia, Chile, Jamaica

- Oceania
    - e.g., Australia, New Zealand, Fiji, Papua New Guinea

- Multiple Regions

- Unclear/Not Specified

Type of evidence source:

- *Primary Research Article:* Peer-reviewed article reporting original research
- *Evidence Synthesis:* Narrative reviews, systematic reviews, scoping reviews, etc.
- *Thesis/Dissertation*

CHARACTERISTICS OF STUDIES INCLUDED

Aim of Study:

Write a short sentence describing the primary aim of the study. To ensure consistency across extractions, use the aim stated in the **final paragraph of the introduction** whenever available. If the introduction does not include a clear aim, refer to the **abstract** as the next best source.

For **theses/dissertations,** locate the aim in the relevant section – typically found in the introduction, state of purpose, or research objectives.

Primary Research Question:

Record the primary research question as stated by the authors. To ensure consistency:

- Use the version found in the **introduction** wherever available.

- If the introduction does not clearly state a research question, refer to the **abstract** as the next best source.
- For **theses/dissertations,** locate the question in the relevant section - typically found in the introduction, state of purpose, or research objectives.
- If no research question is explicitly stated, write: *No research question stated*.

Only report the **primary** research question, even if multiple are listed.

Methodology:
- Qualitative
- Mixed-Methods

Methods:
- Action Research
- Case Study
- Ethnography
- Grounded Theory
- Narrative Inquiry
- Phenomenology
- Qualitative Description
- Other: Please specify
- Unclear/Not Specified
- Not Applicable: Not primary research paper

Data collection:
Indicate the primary method(s) used to collect data to address the study's research question.
- Focus groups
- Interviews
- Survey and/or questionnaires
- Observations
- Archival records
- Other: Please specify
- Not Applicable: Not primary research paper

Context/Setting Description:
Provide a brief but specific description of the context or setting in which the study took place. This may include the professional, educational, clinical, or performance environment relevant to the research.

Examples:
- Rehabilitation
- Sport and Coaching
- Surgical Training

- Research Laboratory
- Clinical Practice

Disciplinary Field:
Identify the primary academic or research discipline the study is situated within, based on the journal, author affiliations, or study focus. Select from the list below or specify if not listed.

- Health Sciences
- Sport Psychology
- Kinesiology
- Other: please specify

Other: Physiotherapy

Aspects of MI investigated:
Provide a brief but specific description of the aspect(s) of motor imagery explored in the study. Focus on the study's primary area of investigation related to motor imagery – this may include its purpose, application, or participant experiences.

Examples:

- Role of motor imagery in learning
- Perceived effectiveness of motor imagery-based interventions
- Experiences of engaging in motor imagery
- Factors influencing motor imagery use in practice
- Athlete's use of motor imagery
- Experiences of implementing motor imagery-based interventions

POPULATION

Population Description:
Provide a brief but specific description of the population studied in the article.

Examples:

- Expert golfers
- Surgeons
- Youth
- Elite athletes

Number of participants:
Record the number of participants per category. Write NA if not a primary research paper or un-specified.

	**Male**	**Female**	**Non-Binary**	**Total (N)**

Count				

Handedness

Record the handedness of participants. Write NA if not a primary research paper or un-specified.

	Left-Handed	Right-Handed	Ambidextrous
Count			

Age

If available, record the age mean and SD. Write NA if not a primary research paper or un-specified.

	Mean	SD
Age		
