# Supplementary material for: Mapping qualitative research on motor imagery: A scoping review
Source: PLoS One. 2026 Apr 29;21(4):e0348064. doi: 10.1371/journal.pone.0348064 (PMC13127901; doi:10.1371/journal.pone.0348064)
Supplement: Attachment — Submitted filename: PLOS_Response_to_Reviewers.pdf [file pone.0348064.s009.pdf]

PONE-D-25-67879

Journal Requirements:

1. Please ensure that your manuscript meets PLOS ONE's style requirements, including those for file naming.

> We have reviewed PLOS ONE's style requirements and trust that we have met all the requirements.

2. We note that there is identifying data in the Supporting Information file S4 File.xlsx. Due to the inclusion of these potentially identifying data, we have removed this file from your file inventory. Prior to sharing human research participant data, authors should consult with an ethics committee to ensure data are shared in accordance with participant consent and all applicable local laws.

> We have carefully reviewed the content of S4 File.xlsx and are confident the revised version does not contain any potentially identifying data. This file provides a comprehensive list of all studies included in the review and is therefore important for transparency and readability of the scoping review. The information contained within the file includes article titles, author information (names and affiliations, which are already publicly available in the published manuscripts), and descriptions of the methodological approaches used in each study. As this manuscript is a scoping review, we do not have access to or report any individual participant data from the included studies. To further ensure that no potentially identifying information is present we removed the following columns from the dataset: *count male, count female, count non-binary, count total, age mean, age SD*. We trust that the remaining information does not contain any potentially identifying data.

3. Please state what role the funders took in the study.

> We have revised our financial disclosure statement to clarify that the funders had no role in study design, data collection and analysis, decision to publish, or preparation of the manuscript.

4. If the reviewer comments include a recommendation to cite specific previously published works, please review and evaluate these publications to determine whether they are relevant and should be cited. There is no requirement to cite these works unless the editor has indicated otherwise.

> Thank for you for your comment. We reviewed the recommendations to cite specific published works from reviewer 1 and agreed that the recommended studies helped to improve the manuscript.

5. Please review your reference list to ensure that it is complete and correct. If you have cited papers that have been retracted, please include the rationale for doing so in the manuscript text, or remove these references and replace them with relevant current references. Any changes to the reference list should be mentioned in the rebuttal letter that accompanies your revised manuscript. If you need to cite a retracted article, indicate the article's retracted status in the References list and also include a citation and full reference for the retraction notice.

We have reviewed the reference list and trust that it is complete and correct. We did not retract any studies from the list of citations.

MAPPING QUALITATIVE RESEARCH ON MOTOR IMAGERY: A SCOPING REVIEW

We would like to thank the reviewers for their feedback and thoughtful comments on our manuscript. We have carefully considered and addressed each comment and believe the manuscript improved as a result. Below you will find your comments (italics) and our responses. Additionally, changes related to the responses have been highlighted in the manuscript. We trust these changes have sufficiently addressed the concerns.

Reviewer #1:

The manuscript "Mapping qualitative research on motor imagery: A scoping review" aims to investigate the extent to which qualitative methods have been used in motor imagery research and to characterize how these approaches have been applied. In this manuscript, the scoping review was conducted in accordance with the JBI methodology. The authors concluded that the literature is heavily weighted toward sport psychology and understanding athletes' use of imagery, with limited attention to how motor imagery is experienced in experimental settings. Moreover, few studies have examined the process of imagery, particularly its role in learning within laboratory contexts. There is a clear absence of qualitative insights into participants' experiences of motor imagery, which represents an important gap in the field. Overall, this study highlights an important gap in motor imagery research.

Introduction

1. *Line 46: Provide references for studies that have reported conflicting results.*

Thank you for your suggestion. We have modified the manuscript to address this comment by incorporating concrete examples that illustrate the mixed

findings regarding the efficacy of motor imagery. In the revised manuscript we cite Ietswaart et al. (2011) as an example of a study reporting no significant benefit of motor imagery in stroke recovery, alongside Page et al. (2005) demonstrating the opposite effect.

Ietswaart, M., Johnston, M., Dijkerman, H. C., Joice, S., Scott, C. L., MacWalter, R. S., & Hamilton, S. J. C. (2011). Mental practice with motor imagery in stroke recovery: randomized controlled trial of efficacy. *Brain: A Journal of Neurology*, *134*(Pt 5), 1373–1386. https://doi.org/10.1093/brain/awr077

Page, S. J., Levine, P., & Leonard, A. C. (2005). Effects of mental practice on affected limb use and function in chronic stroke. *Archives of Physical Medicine and Rehabilitation*, *86*(3), 399–402. https://doi.org/10.1016/j.apmr.2004.10.002

2. *Line 77: You may also include the following articles:*

Parimi, F., Abdoli, B., Ramezanzade, H., & Aghdaei, M. (2024). The effect of internal and external imagery on learning badminton long serve skill: The role of visual and audiovisual imagery. PLOS ONE, 19(9), e0309473.

Ramezanzade, H., Badicu, G., Cataldi, S., Parimi, F., Mohammadzadeh, S., Mohamadtaghi, M., & Greco, G. (2023). Sonification of motor imagery in the basketball jump shot: Effect on muscle activity amplitude. Applied Sciences, 13(3), 1495.

Thank you for the recommended articles. We have revised the manuscript to include both.

3. *Lines 84–91: The concept of qualitative research should be explicitly introduced in the Introduction. Clarify what is meant by qualitative methods in this context.*

We agree with the reviewer that providing a definition of qualitative research in the introduction would improve clarity. In response, we have added a definition drawing on Creswell and Poth (2024), defining qualitative research as an approach aimed at understanding and exploring human experience through the collection and analysis of non-numerical data, where researchers construct meaning from the data, generating themes or patterns.

Creswell, J. W., & Poth, C. N. (2024, November 7). *Qualitative Inquiry and Research Design*. SAGE Publications Ltd.

https://uk.sagepub.com/en-gb/eur/qualitative-inquiry-and-research-design/book266033

Design

4. *Line 115: Why was no outcome criterion included? Doesn't the participants' level and type of experience (e.g., children vs. adults, healthy vs. disabled) affect the quality of imagery? Please justify this decision.*

Thank you for your thoughtful question. The purpose of this scoping review was to map how qualitative methods have been used in motor imagery literature, rather than to synthesize findings about the *experience* of motor imagery itself. In accordance with guidance from JBI, our research was best addressed through a scoping review designed to examine the extent and nature of methodological approaches in the existing literature. Had our objective been to explore participants' experiences of motor imagery, JBI recommendations include a distinct research question on experience using a qualitative systematic review approach. Therefore, in line with our stated objective, we excluded analyses of participant experience from the present review.

Search methods

5. *Line 130: What were the criteria for excluding articles? Please specify.*

Thank you for your suggestion to be more explicit about the exclusion criteria. While these criteria were clearly specified in the cited pre-registration of the scoping review protocol, we agree that articulating them more explicitly in the manuscript strengthens clarity and transparency.

As noted in the manuscript, there were no exclusion criteria based on participant characteristics. Studies were excluded if they focused on action observation or combined action observation and motor imagery (AOMI). We also excluded studies involving imagery that was not explicitly motor in nature (e.g., visual-only, auditory, etc.). Studies employing exclusively quantitative methodologies were excluded, whereas mixed-methods studies with a qualitative component were included. All non-English literature was excluded.

We have revised the manuscript to more clearly specify the exclusion criteria related to both concept and context.

Results

6. *Line 167: What were the inclusion criteria? Clarify explicitly.*

   Thank you for your question. The inclusion criteria are described in the Design subsection of the Methods. However, we recognize that using the term *Eligibility Criteria* may have caused some confusion. To improve clarity and consistency, we have revised the manuscript to use the label *Inclusion Criteria,* ensuring that readers can easily locate and interpret the criteria when they are referenced in the Results section.

<u>Reviewer #2:</u>

This is a well-executed scoping review that addresses an important gap in motor imagery research. The authors systematically map the use of qualitative methods in this field and identify meaningful gaps, particularly the absence of qualitative inquiry into motor imagery experiences during experimental learning contexts. The manuscript is clearly written, methodologically sound, and makes a compelling case for expanding qualitative approaches beyond sport psychology into rehabilitation and fundamental motor learning research.

Limited Critical Analysis of Methodology Quality

While you note that 51% of studies didn't report a specific design, you don't systematically appraise the quality of included studies. For a scoping review, this is acceptable, but you could strengthen the manuscript by:

1. *Discussing patterns in methodological rigor beyond just reporting design*
2. *Providing examples of well-designed versus poorly-designed studies*
3. *Commenting on sampling adequacy, data saturation, or analytical depth across studies*

Thank you for your thoughtful and constructive comment. The primary aim of this scoping review was to map the extent, range, and characteristics of qualitative research within the motor imagery literature. At the outset of the review, the degree to which qualitative approaches had been used in this field was unknown. As such, our research questions were intentionally framed to identify and characterize the presence and types of qualitative inquiry, rather than to systematically appraise methodological quality. In line with scoping review guidance from JBI, we did not conduct a formal critical appraisal, as this was beyond the stated objectives of the review. Regarding sampling

adequacy, data saturation, and analytical rigor, we recognize that these are commonly referenced markers of quality. However, such criteria often emerge from post-positivist epistemological traditions and may not align with in the assumptions or evaluative standards of all qualitative paradigms (e.g., interpretivist, constructivist, or critical approaches). Given that one contribution of our scoping review is to highlight the need for and value of diverse qualitative approaches within motor imagery research, we are cautious not to impose a single set of quality markers that could inadvertently reproduce the dominant paradigmatic assumptions already shaping the field. Instead, we have focused on describing patterns in methods, allowing readers to interpret rigor within the appropriate epistemological context.

Insufficient Exploration of *Why* These Gaps Exist

You identify important gaps but don't deeply explore their causes:

4. *Why has sport psychology embraced qualitative methods while motor learning hasn't?*
5. *Are there epistemological barriers in experimental psychology/neuroscience?*
6. *What institutional or funding structures might discourage qualitative work in laboratories?*
7. *Do researchers lack training in qualitative methods?*

Thank you for raising this important point. We agree that further consideration of why these gaps exist strengthens the manuscript. In response, we have expanded the introduction to suggest that one potential contributor is the dominance of post-positivist paradigmatic assumptions within motor imagery research, which shape what is considered knowable, measurable, and valued within the field. Such assumptions may create epistemological barriers within experimental psychology and neuroscience, influence funding priorities, and shape researcher training in ways that constrain the uptake of qualitative approaches.

At the same time, we recognize that the structural and paradigmatic factors underlying this gap are complex and cannot be adequately addressed within the present scoping review. A more in-depth discussion is beyond the aims of this paper. We are currently preparing a separate manuscript that engages this question directly and provides a more detailed examination of these paradigmatic influences.

Weak Recommendations for Future Research

Your conclusion calls for "clearer reporting of qualitative designs and more in-depth exploration" but doesn't provide concrete guidance:

8. *What specific qualitative approaches would be most valuable for laboratory studies?*

9. *How might researchers integrate qualitative and quantitative methods in motor learning experiments?*

10. *What questions should future qualitative research prioritize?*

11. *Are there practical barriers to overcome?*

Thank you for your thoughtful question. We agree that our original recommendations for future research were broad and could benefit from greater specificity. In response to question 8, we do not believe that a single qualitative approach is most valuable for laboratory studies. The central issue identified in this scoping review is that participant experience has largely been excluded from the field. Accordingly, our primary recommendation is to broadly increase the integration of qualitative approaches. The most suitable methodology will depend on the specific research question, context, theoretical framework, and the researcher's paradigmatic positioning.

That said, we agree that offering a more concrete example strengthens the manuscript. We have therefore revised the recommendations for future research to propose the use of an exploratory sequential mixed methods design (Creswell, 2024). This approach is particularly appropriate when a phenomenon is not yet well understood and when existing quantitative instruments may not adequately capture the phenomenon. In this design, researchers would first use qualitative methods to explore how individuals experience motor imagery. Insights from this phase could then inform the refinement or development of motor imagery measures grounded in participants' experiences. These refined tools would subsequently be evaluated in larger-scale quantitative learning experiments. In addition, drawing on the reviewer's earlier comment, we've added the recommendation of systematic appraisal of the existing qualitative literature to address questions of methodological quality and rigor.

We further expanded our discussion to acknowledge structural barriers to embracing more pluralistic forms of knowledge production, particularly given the dominance of post-positivism in the field. Addressing these gaps requires effort across the research ecosystem: (1) institutions must provide training in qualitative theory and methodology; (2) journals must create space for qualitative inquiry within motor imagery and related disciplines; and (3) funding agencies must support research that investigates the experiential dimensions of inherently covert phenomena, such as motor imagery.

Creswell, J. W. (2024, December 11). *A Concise Introduction to Mixed Methods Research*. SAGE Publications Inc. https://us.sagepub.com/en-us/nam/a-concise-introduction-to-mixed-methods-research/book266037

Abstract Could Be More Specific

12. *The abstract mentions limited attention to how motor imagery is experienced in experimental settings but doesn't quantify this. Since only 1/39 studies examined learning, state this explicitly in the abstract.*

   Thank you for your comment. We agree that highlighting this finding in the abstract is important. However, this statistic is already explicitly stated. The final sentence of the Results section of the abstract reads: “The predominant focus was on how specific populations use motor imagery (*n* = 27; 69.2%), while only one study (2.6%) investigated learning through motor imagery.” We believe that this clearly communicates that only 1 of the 39 studies (2.6%) examined learning, thereby quantifying the limited attention to motor imagery in experimental learning contexts.

Introduction Could Strengthen the "So What"

While you explain why qualitative research matters, you could more explicitly connect this to practical outcomes. For instance:

13. *How might misunderstanding participants' imagery experiences lead to failed interventions?*
14. *Are there examples where quantitative findings might be reinterpreted with qualitative insight?*
15. *What rehabilitation outcomes might improve with better understanding of patient experiences?*

We agree with the reviewer that more explicitly linking the need for qualitative research to practical outcomes strengthens the manuscript. For question 13, we are currently unable to point to specific examples in which misunderstanding participants’ imagery experiences has led to failed interventions. This is largely because participants’ experiential accounts of motor imagery in experimental and applied contexts remain underexplored. In response to questions 14 and 15, we have revised the Introduction to more clearly articulate the practical implications of this gap. Specifically, we now state that because motor imagery functions as the central experimental variable in imagery-based learning studies, unexamined variation in how imagery is experienced may directly influence conclusions about its efficacy and proposed mechanisms of learning. Consequently, translation to applied contexts such as rehabilitation and sport may rest more on researchers’ assumptions about imagery processes than on participants’ experiences of motor imagery. This disconnect has the potential to influence design, uptake and overall

effectiveness in these contexts. We have also expanded the Conclusion to emphasize that systematic engagement with qualitative inquiry could strengthen the application of motor imagery in rehabilitation by centering intervention design and evaluation on patient experience. Grounding motor imagery practice in how individuals engage with and interpret imagery may enable researchers and clinicians to develop more responsive, meaningful, and ultimately more effective interventions.

Results Section Organization

Table 1 is comprehensive but dense. Consider:

16. *Breaking it into two tables (study characteristics vs. research focus)*
17. *Using visualization (perhaps a bar chart showing disciplinary distribution)*
18. *Highlighting key percentages in the text before referring to the table*

Thank you for your recommendations. After carefully reviewing the Results section, we maintain that although Table 1 is dense, it remains readable and easy to navigate. Dividing it into two separate tables may increase the cognitive load required by readers to move back and forth between tables and synthesize related information. We have also ensured that key percentages are explicitly highlighted in the text prior to directing readers to the table. If there are specific instances where additional clarification would be helpful, we would be grateful for further guidance and are happy to revise accordingly. That said, we agree that a visual summary may strengthen communication of our key finding that the majority of qualitative motor imagery research is situated within sport psychology. In response, we have added Fig 2, a pie chart illustrating the disciplinary distribution of included studies, to enhance clarity and accessibility of this result.

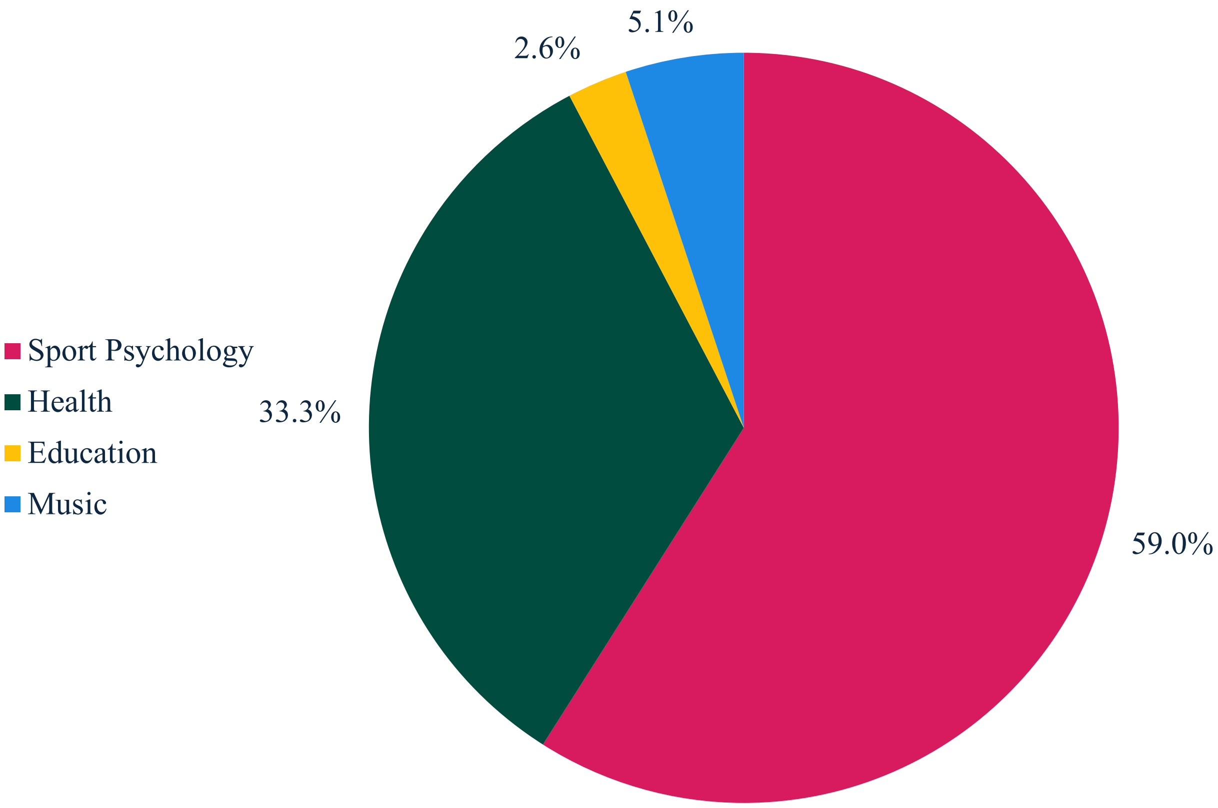

Fig 2. Disciplinary distribution of included studies.

Missing Discussion of Mixed Methods

You note that 20.5% of studies used mixed methods but don't discuss:

19. *How were qualitative and quantitative approaches integrated?*

20. *Did mixed methods studies yield different insights than purely qualitative ones?*
21. *Should mixed methods be the recommended approach for motor learning research?*

Thank you for these thoughtful questions. The primary objective of this scoping review was to map the extent to which qualitative approaches have been used in motor imagery research and to characterize those qualitative components. Accordingly, our data extraction focused specifically on the qualitative elements of the included studies. We did not extract or analyze information regarding how qualitative and quantitative components were integrated, nor did we compare outcomes between mixed methods and purely qualitative designs. As such, we are unable to comment on whether mixed methods studies yielded substantively different insights or on the specific nature of integration strategies employed across studies. Regarding whether mixed methods should be recommended for motor learning research, we do not argue that mixed methods are superior. Rather, in response to an earlier reviewer comment, we now suggest that an exploratory sequential mixed methods design may be particularly valuable in contexts where experiential dimensions of motor imagery are not well understood and existing quantitative tools may lack sensitivity. This recommendation is intended as one possible direction for future research but is not a prescriptive standard for the field.

Limitations Section Feels Perfunctory

Your limitations (English-only, short timeline, limited grey literature) are fairly standard. Consider discussing:

22. *Potential publication bias (are qualitative motor imagery studies less likely to be published?)*
23. *Whether your search terms might have missed relevant work*
24. *Challenges in defining "qualitative" when many quantitative studies collect self-report data*

Thank you for your suggestions. We agree that publication bias may limit the amount of qualitative research available in motor imagery literature. However, we would consider this a feature of the broader research landscape rather than a limitation of our search strategy or the scoping review methodology itself. If qualitative studies are underrepresented in published literature, this would help explain the small number identified, but it does not reflect a procedural shortcoming of the review. That said, it is possible that our search terms may have missed relevant studies. In response, we have added this as a limitation in the manuscript. Nonetheless, our search strategy was developed in consultation with a health sciences librarian and are confident that the strategy was appropriately comprehensive for the purposes of this scoping review. Finally, we included in the limitations that for this review, we did not operationalize self-report data alone as constituting qualitative research. While this may in some cases be

qualitative *data,* the paradigmatic positioning, theoretical framework, and methodology is lacking, thus self-report data was not sufficient for classification as qualitative research within the current scoping review.
