# Supplementary material for: Mapping qualitative research on motor imagery: A scoping review
Source: PLoS One. 2026 Apr 29;21(4):e0348064. doi: 10.1371/journal.pone.0348064 (PMC13127901; doi:10.1371/journal.pone.0348064)
Supplement: Attachment — Submitted filename: Re_.docx [file pone.0348064.s006.docx]

**Re:** **Mapping qualitative research on motor imagery: A scoping review**

**General comments**

The manuscript “Mapping qualitative research on motor imagery: A scoping review” aims to investigate the extent to which qualitative methods have been used in motor imagery research and to characterize how these approaches have been applied. In this manuscript, the scoping review was conducted in accordance with the JBI methodology. The authors concluded that the literature is heavily weighted toward sport psychology and understanding athletes’ use of imagery, with limited attention to how motor imagery is experienced in experimental settings. Moreover, few studies have examined the process of imagery, particularly its role in learning within laboratory contexts. There is a clear absence of qualitative insights into participants’ experiences of motor imagery, which represents an important gap in the field. Overall, this study highlights an important gap in motor imagery research.

Below are some suggestions that should be considered by the authors:

**Specific comments**

**Introduction**

Line 46: Provide references for studies that have reported conflicting results.

Line 77: You may also include the following articles:

Parimi, F., Abdoli, B., Ramezanzade, H., & Aghdaei, M. (2024). The effect of internal and external imagery on learning badminton long serve skill: The role of visual and audiovisual imagery. PLOS ONE, 19(9), e0309473.

Ramezanzade, H., Badicu, G., Cataldi, S., Parimi, F., Mohammadzadeh, S., Mohamadtaghi, M., & Greco, G. (2023). Sonification of motor imagery in the basketball jump shot: Effect on muscle activity amplitude. Applied Sciences, 13(3), 1495.

Lines 84–91: The concept of qualitative research should be explicitly introduced in the Introduction. Clarify what is meant by qualitative methods in this context.

**Design**

Line 115: Why was no outcome criterion included? Doesn’t the participants’ level and type of experience (e.g., children vs. adults, healthy vs. disabled) affect the quality of imagery? Please justify this decision.

Search methods

Line 130: What were the criteria for excluding articles? Please specify.

**Results**

Line 167: What were the inclusion criteria? Clarify explicitly.
