# Supplementary material for: Mapping qualitative research on motor imagery: A scoping review
Source: PLoS One. 2026 Apr 29;21(4):e0348064. doi: 10.1371/journal.pone.0348064 (PMC13127901; doi:10.1371/journal.pone.0348064)
Supplement: Attachment — Submitted filename: Review_ Mapping qualitative research on motor imagery.docx [file pone.0348064.s007.docx]

This is a well-executed scoping review that addresses an important gap in motor imagery research. The authors systematically map the use of qualitative methods in this field and identify meaningful gaps, particularly the absence of qualitative inquiry into motor imagery experiences during experimental learning contexts. The manuscript is clearly written, methodologically sound, and makes a compelling case for expanding qualitative approaches beyond sport psychology into rehabilitation and fundamental motor learning research.

**Areas for Improvement**

**1. Limited Critical Analysis of Methodology Quality** While you note that 51% of studies didn't report a specific design, you don't systematically appraise the quality of included studies. For a scoping review, this is acceptable, but you could strengthen the manuscript by:

- Discussing patterns in methodological rigor beyond just reporting design
- Providing examples of well-designed versus poorly-designed studies
- Commenting on sampling adequacy, data saturation, or analytical depth across studies

**2. Insufficient Exploration of *Why* These Gaps Exist** You identify important gaps but don't deeply explore their causes:

- Why has sport psychology embraced qualitative methods while motor learning hasn't?
- Are there epistemological barriers in experimental psychology/neuroscience?
- What institutional or funding structures might discourage qualitative work in laboratories?
- Do researchers lack training in qualitative methods?

**3. Weak Recommendations for Future Research** Your conclusion calls for "clearer reporting of qualitative designs and more in-depth exploration" but doesn't provide concrete guidance:

- What specific qualitative approaches would be most valuable for laboratory studies?
- How might researchers integrate qualitative and quantitative methods in motor learning experiments?
- What questions should future qualitative research prioritize?
- Are there practical barriers to overcome?

**4. Abstract Could Be More Specific** The abstract mentions limited attention to how motor imagery is experienced in experimental settings but doesn't quantify this. Since only 1/39 studies examined learning, state this explicitly in the abstract.

**5. Introduction Could Strengthen the "So What"** While you explain why qualitative research matters, you could more explicitly connect this to practical outcomes. For instance:

- How might misunderstanding participants' imagery experiences lead to failed interventions?
- Are there examples where quantitative findings might be reinterpreted with qualitative insight?
- What rehabilitation outcomes might improve with better understanding of patient experiences?

**6. Results Section Organization** Table 1 is comprehensive but dense. Consider:

- Breaking it into two tables (study characteristics vs. research focus)
- Using visualization (perhaps a bar chart showing disciplinary distribution)
- Highlighting key percentages in the text before referring to the table

**7. Missing Discussion of Mixed Methods** You note that 20.5% of studies used mixed methods but don't discuss:

- How were qualitative and quantitative approaches integrated?
- Did mixed methods studies yield different insights than purely qualitative ones?
- Should mixed methods be the recommended approach for motor learning research?

**8. Limitations Section Feels Perfunctory** Your limitations (English-only, short timeline, limited grey literature) are fairly standard. Consider discussing:

- Potential publication bias (are qualitative motor imagery studies less likely to be published?)
- Whether your search terms might have missed relevant work
- Challenges in defining "qualitative" when many quantitative studies collect self-report data
